# Supplementary material for: Genome-Wide Identification of the Lectin Receptor-like Kinase Gene Family in Avena sativa and Its Role in Salt Stress Tolerance
Source: Int J Mol Sci. 2024 Nov 27;25(23):12754. doi: 10.3390/ijms252312754 (PMC11641407; doi:10.3390/ijms252312754)
Supplement: Supplementary file 1 [file ijms-25-12754-s001.zip › ijms-3307196-supplementary/Supplementary Figures.pdf]

Gui Xiong et al. Genome-Wide Identification of LecRLKs Gene Family in *Avena sativa*. Supplementary Figures S1–S4.

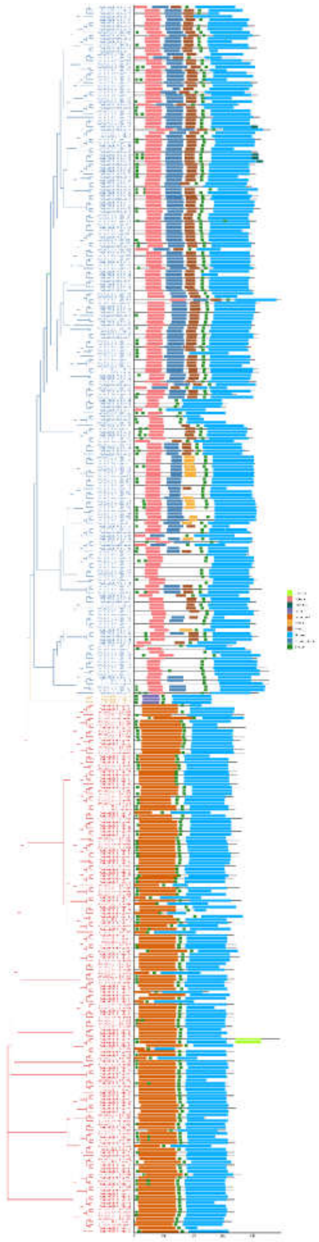

Figure S1. Phylogenetic relationship of protein sequences of G-, L- and C-type AsaLecRLKs in *Avena sativa* using the maximum-likelihood method. G-type AsaLecRLKs contain bulb lectin domain, S-locus glycoprotein domain, and PAN domain at the N-terminus and protein kinase domain and DUF3403 domain at the C-terminus; L-type AsaLecRLKs contain the legume lectin domain at the N-terminus and protein kinase domain and adh_short domain at the C-terminus; C-type AsaLecRLK contains the calcium-binding lectin domain at the N-terminus and protein kinase domain at the C-terminus.

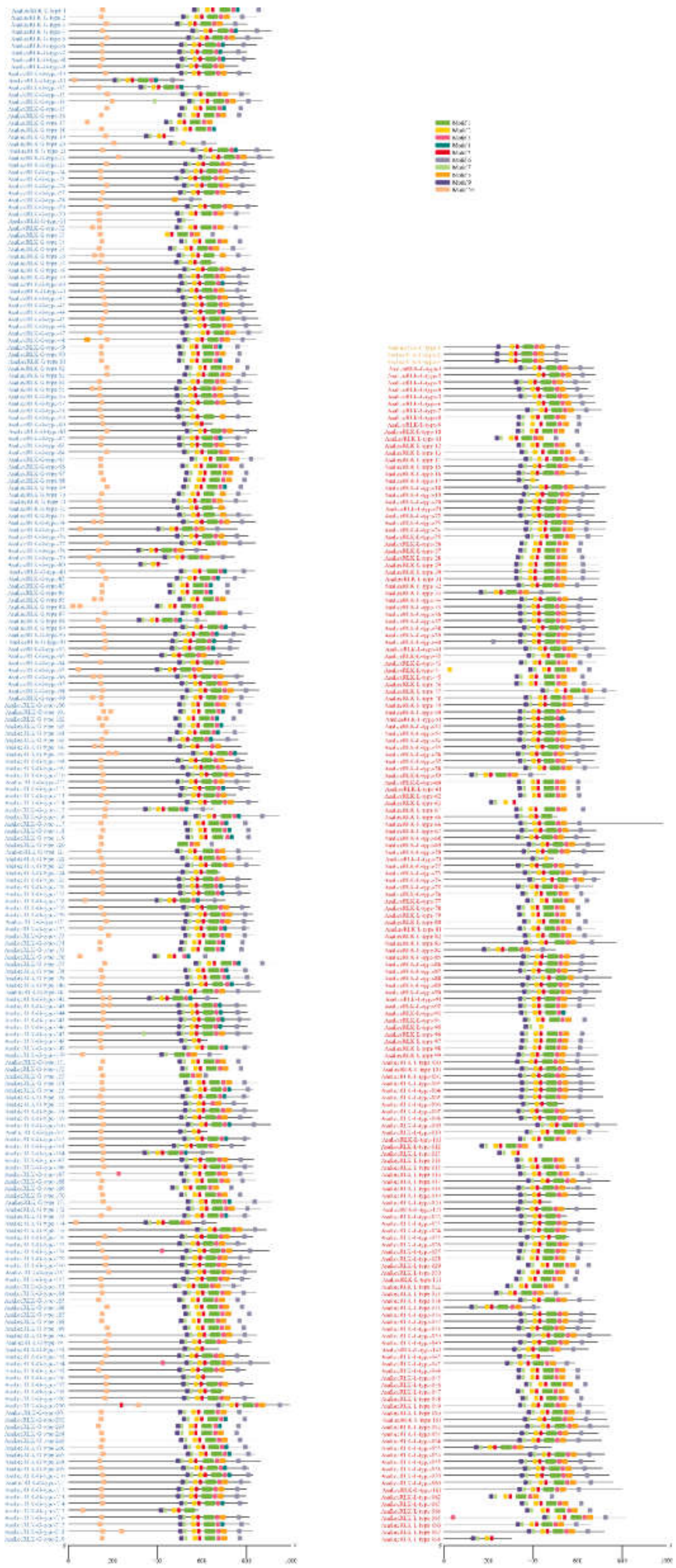

Figure S2. Ten motifs detected in protein sequences of G-, L- and C-type AsaLecRLKs in *Avena sativa* using MEME online tools.

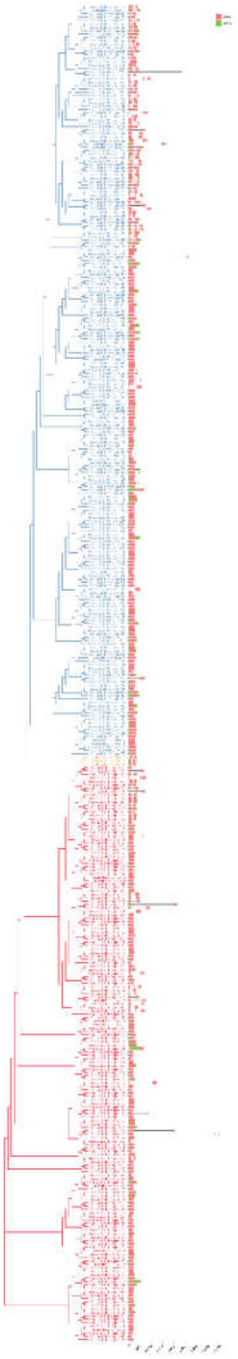

Figure S3. Phylogenetic relationships of *LecRLK* gene structure of *Avena sativa* using the maximum-likelihood method. The number of *LecRLK* gene exons of *A. sativa* range from 1 to 9, most (215 out of 390) genes have a single exon. Intron length varies from 61 bp (AsaLecRLK-G-type-106) to 2041 bp (AsaLecRLK-L-type-27).

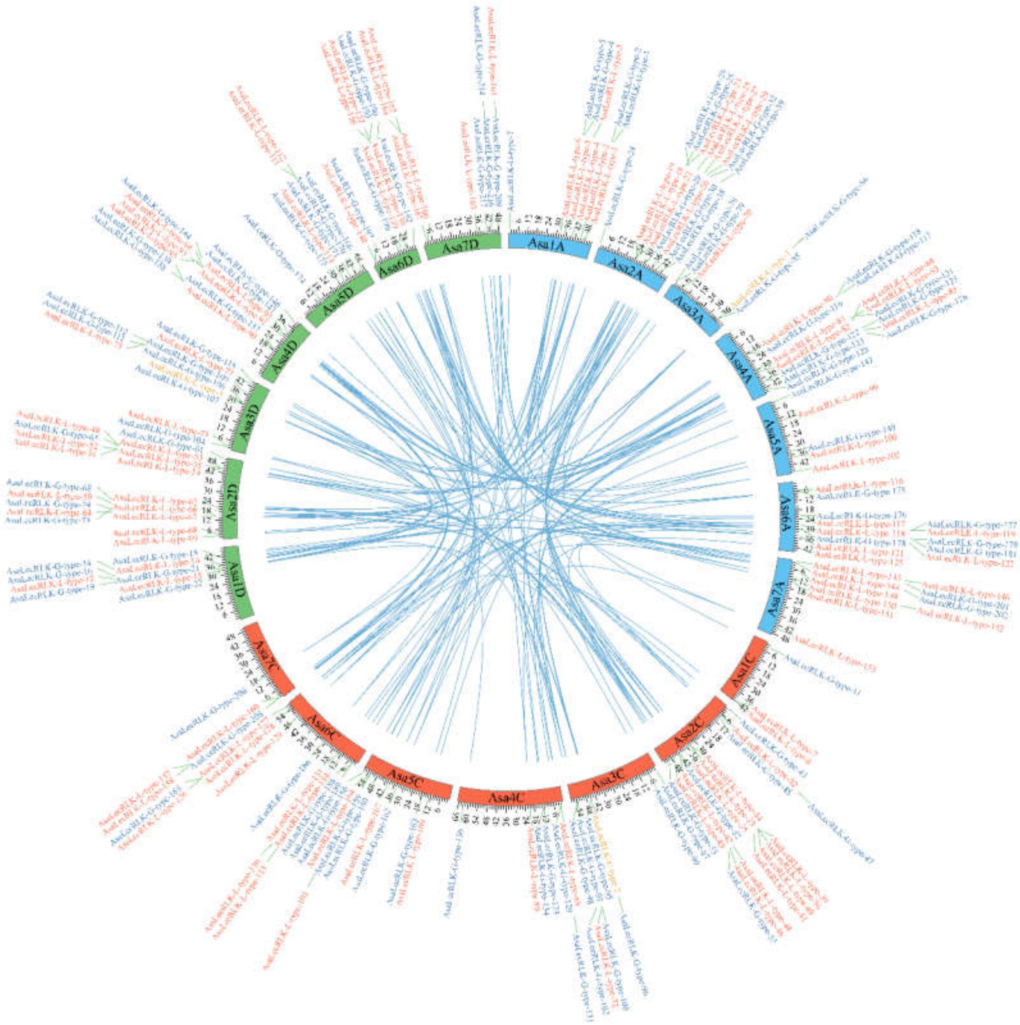

Figure S4. Gene duplications of *LecRLKs* gene family in *Avena sativa*. Blue lines (centre) indicate segmental duplicated gene pairs in *LecRLKs*.
